# Substitution of adeno-associated virus Rep protein binding and nicking sites with human Chromosome 19 sequences

**DOI:** 10.1186/1743-422X-7-218

**Published:** 2010-09-08

**Authors:** Victor J McAlister, Roland A Owens

**Affiliations:** 1 Laboratory of Molecular and Cellular Biology, National Institute of Diabetes and Digestive and Kidney Diseases, National Institutes of Health, Department of Health and Human Services, Bethesda, Maryland 20892, USA

## Abstract

**Background:**

Adeno-associated virus type 2 (AAV2) preferentially integrates its DNA at a ~2 kb region of human chromosome 19, designated *AAVS1 *(also known as *MBS85*). Integration at *AAVS1 *requires the AAV2 replication (Rep) proteins and a DNA sequence within *AAVS1 *containing a 16 bp Rep recognition sequence (RRS) and closely spaced Rep nicking site (also referred to as a terminal resolution site, or *trs*). The AAV2 genome is flanked by inverted terminal repeats (ITRs). Each ITR contains an RRS and closely spaced *trs*, but the sequences differ from those in *AAVS1*. These ITR sequences are required for replication and packaging.

**Results:**

In this study we demonstrate that the *AAVS1 *RRS and *trs *can function in AAV2 replication, packaging and integration by replacing a 61 bp region of the AAV2 ITR with a 49 bp segment of *AAVS1 *DNA. Modifying one or both ITRs did not have a large effect on the overall virus titers. These modifications did not detectably affect integration at *AAVS1*, as measured by semi-quantitative nested PCR assays. Sequencing of integration junctions shows the joining of the modified ITRs to *AAVS1 *sequences.

**Conclusions:**

The ability of these *AAVS1 *sequences to substitute for the AAV2 RRS and *trs *provides indirect evidence that the stable secondary structure encompassing the *trs *is part of the AAV2 packaging signal.

## Background

Adeno-associated viruses (AAVs) are mammalian parvoviruses that typically require a helper virus, such as an adenovirus or herpesvirus for productive replication [1]. Multiple AAV serotypes have been described. The most detailed information is available for AAV serotype 2 (AAV2), the first human isolate. In the absence of helper virus, AAV2 preferentially integrates into a region of human chromosome 19 (19q13.4ter) referred to as Adeno-Associated Virus Site 1, or *AAVS1 *[2-5]. In cultured cells infected with a high multiplicity of virus, approximately 70% of integration events have been reported to occur at *AAVS1 *[6-9]. Site-specific integration would be useful for many gene therapy applications, but most recombinant AAV vectors do not utilize the ability of AAV2 to integrate site-specifically [10].

**Figure 1:**
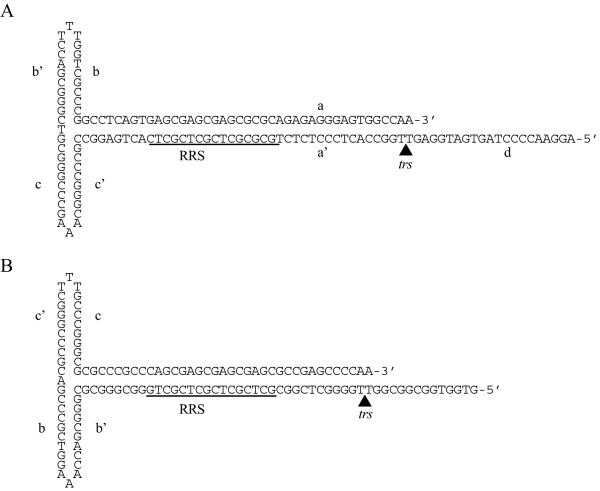
**Structure of the wild-type AAV2 and modified ITR**. (A) The AAV2 ITR is composed of seven regions, a, a', b, b', c, c' and d, and forms an extensive secondary structure. The b, b', c and c' regions exist in two configurations, "flip" and "flop." The wild type ITR is shown in the flip configuration. (B) A modified version of the ITR, in which the a, a' and d regions are replaced with *AAVS1 *DNA, was designed in the flop configuration.

AAV2 has a 4.7 kb single-stranded DNA genome flanked at each end by 145 base inverted terminal repeats (ITRs) [11]. The ITRs are required for viral replication and packaging and occur in two forms, referred to as "flip" and "flop" (Fig. 1) [12-14]. The flip and flop conformations are a consequence of the rolling hairpin mechanism of AAV2 replication [15]. AAV2 contains two open reading frames that occupy most of the remainder of the genome. The right open reading frame encodes three capsid proteins, VP1, 2 and 3 [16,17]. The left open reading frame encodes the four nonstructural replication proteins [18,19]. The large replication proteins, Rep78 and 68, possess sequence-specific DNA binding, ATPase, helicase and sequence-specific, strand-specific endonuclease activities [20-23]. Rep 68 or 78 are required for AAV2 replication [24,25].

Each ITR forms a double hairpin, or "T" shaped structure (Fig. 1). The ITRs contain a 16 bp, double-stranded, Rep recognition sequence (RRS), consisting of four imperfect GCTC repeats [22,26], and a Rep68/78 nicking site, or terminal resolution site (*trs*) [20]. Rep68/78 binds to the RRS as a multimer [27,28] and unwinds the DNA [29], allowing the formation of a specific secondary structure at the *trs *[30,31]. Rep78/68 nicks one strand of the DNA between the two adjacent thymine residues in the *trs *[20,32], creating a free 3'-end which is required for replication of the end of the AAV2 genome. Several lines of evidence indicate that replication and packaging are coupled [33]. Capsid interactions have been observed with all four Rep proteins [34] and single-stranded AAV2 DNA also does not accumulate in the absence of capsids [35] or Rep 52/40 [25]. The helicase function of Rep52/40 is believed to be required to insert the replicated DNA into the pre-formed capsids and the DNA is inserted from the 3'-end [36].

*AAVS1 *contains a RRS [26] and a closely spaced *trs *[37], an arrangement that is thought to be unique in the human genome [5,38]. A 33 bp region of *AAVS1 *encompassing the RRS and *trs *is sufficient to target integration of wild-type AAV2 into an episome [39-41]. Rep68/78 is also required for AAV2 integration at *AAVS1 *on chromosome 19 [8,42,43]. Sequence data are available for a number of AAV2-*AAVS1 *junctions [2,40,44]. AAV2 junctions within *AAVS1 *have been shown to occur only on one side of the *AAVS1 trs*. *AAVS1 *DNA also serves as a Rep68/78-dependent, unidirectional origin of replication *in vitro *[37]. These observations are consistent with DNA synthesis from the nicking site being part of the integration mechanism.

More recent reports have shown the encapsidation of *AAVS1 *sequences as a byproduct of AAV2 production [45]. Encapsidation of sequences containing a cryptic RRS/*trs *combination found at the p5 promoter of AAV2 [46-48], as well as encapsidation of prokaryotic sequences linked to AAV2 ITRs have also been reported when a plasmid-based packaging system was used [49]. These observations suggest degeneracy in the sequences that can be used as AAV2 replication and packaging signals.

In this report we have extended the existing homology between the ITR and *AAVS1 *by replacing 61 bp of sequence containing the RRS and *trs *with 49 bp of *AAVS1 *sequence containing the *AAVS1 *RRS and *trs*. We find that AAV2 modified in this way can replicate, package and integrate similar to the wild-type virus.

## Results

### Replacement of the AAV2 ITR RRS and *trs *with chromosome 19 DNA

The AAV2 ITR forms an extensive secondary structure and is composed of seven regions, a, a', b, b', c, c' and d (Fig. 1A). The ITRs in the AAV2 infectious clone pSub201(-) are flanked by Pvu II and Xba I sites. These sites were used to replace the ITRs with a synthetic ITR obtained from a commercial supplier. The a, a' and d regions are replaced with *AAVS1 *DNA in the modified ITR (Fig. 1B).

### Packaging and replication

**Figure 2:**
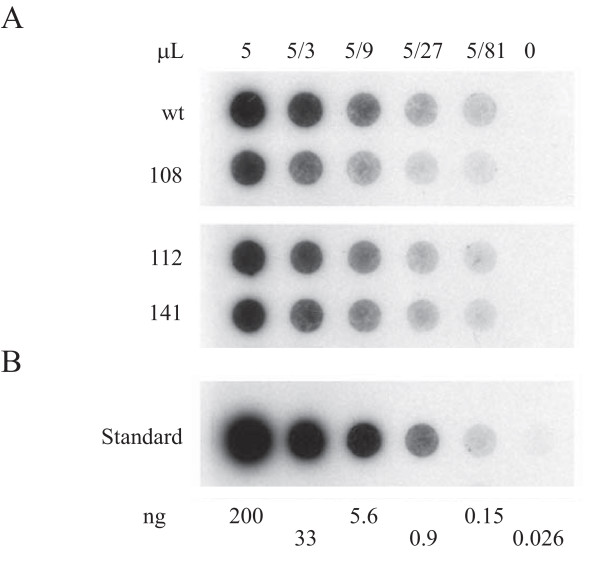
**Packaging comparisons between the wild-type and modified viruses**. (A) Dot blot analysis of 3-fold serial dilutions of nuclease-resistant AAV2 DNA made using pSub201(-) (wt), pVM108 (108) pVM112 (112) or pVM141 (141). The first dot in each row has the nuclease-resistant DNA obtained from 5 μl of virus supernatant. (B) Standards. The indicated amount of linearized and denatured pSub201(-) was applied to the same membrane. All samples were probed with random primer radiolabeled AAV2 DNA.

A two plasmid system was used to package AAV2. One plasmid contained the AAV2 genome with wild-type and/or modified ITRs. The second plasmid expressed adenovirus genes that promote AAV replication. The two plasmids were used to co-transfect a human embryonic kidney cell line expressing the adenovirus E1 gene (Stratagene HEK293 cells). The cells were later lysed to collect the packaged virus. During this procedure DNA that was not packaged as virus was removed by nuclease treatment. Hybridization analysis was used to measure the amount of virus DNA that was resistant to nuclease treatment. Virus proteins were removed by protease treatment prior to applying the virus DNA to the membrane. To calibrate the number of genomes that were packaged, various dilutions of a linearized AAV2 plasmid were denatured and applied to the membrane. For the virus titer determination, the membrane was hybridized to random primer radiolabeled AAV2 DNA from the wild-type AAV2 plasmid. The results are shown in Fig. 2. Three-fold dilutions of nuclease-resistant DNA were applied to the membrane in part A of the figure. The row labeled wt is nuclease-resistant AAV2 DNA derived from cells co-transfected with the adenovirus helper plasmid and pSub201(-). The virus DNA in the row labeled 108 was made using pVM108. pVM108 is a pSub201(-) derivative in which both ITRs are replaced with the modified sequence. The virus DNA in rows labeled 112 and 141 was made from pVM112 and pVM141, respectively. pVM112 and pVM141 are pSub201(-) derivatives in which only one ITR is replaced with the modified sequence. pVM112 and pVM141 have an additional 49 bp of *AAVS1 *sequence (beginning 1509 bp from the *AAVS1 trs*) between the right ITR and the cap gene. Approximately the same amount of AAV2 was packaged with genomes containing wild-type, or one or two modified ITRs. The titers are in the range of 10^12 ^packaged virus genomes per 75 cm^2 ^flask of cells.

**Figure 3:**
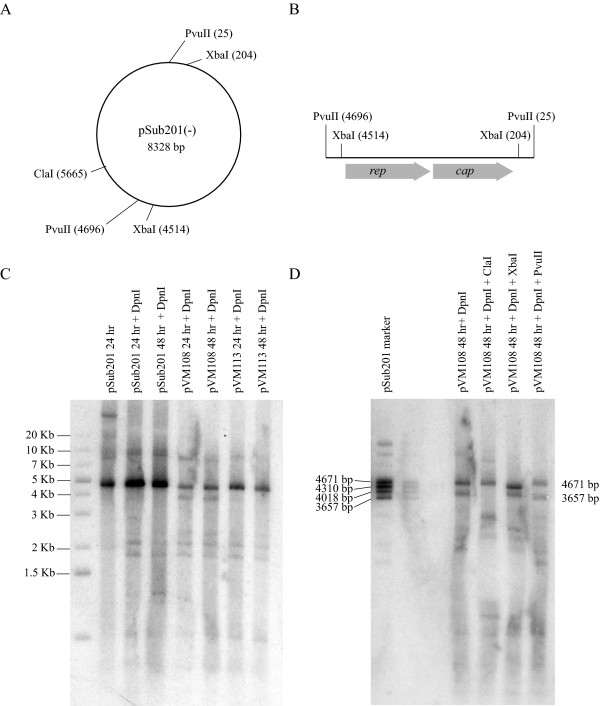
**Viral DNA replication**. (A) Restriction map of pSub201(-). (B) Location and orientation of the AAV2 genome in pSub201(-). (C) Southern blot of genomic DNA from cells cotransfected with an adenovirus helper plasmid and the indicated AAV2 plasmids. The membrane was probed with random primer ^32^P-labeled, linearized pSub201(-). The molecular weight marker is homologous to the backbone portion of pSub201. (D) Southern blot of genomic DNA from cells cotransfected with an adenovirus helper plasmid and pVM108. The membrane was probed with random primer labeled pSub201. The pSub201(-) marker was made by running XbaI and PvuII digests in the same lane.

Virus DNA replication in plasmid-transfected cells was analyzed by using the Southern blotting procedure. A map of the pSub201(-) vector used to make AAV2 containing two wild type ITRs is shown in Figure 3A. As shown in Figure 3B, the AAV2 genome is located within a PvuII fragment of the vector. The vector maps for pVM108 (both ITRs modified) and pVM113 (left ITR modified) are similar to pSub201(-). In all three vectors the ITRs are located between the PvuII and XbaI sites. Figure 3C is a Southern analysis of AAV2 replication in cells transfected with pSub201(-), pVM108 or pVM113. The cells were cotransfected with an adenovirus helper plasmid. Equal amounts of genomic DNA were loaded in each of the sample lanes. The membrane was probed with randomly labeled pSub201(-) DNA. Replicated and input plasmid DNA were differentiated by DpnI treatment, which only digests bacterially methylated DNA. The major band of replicated DNA in each of the sample lanes is approximately 5 Kb, which corresponds to size of the AAV2 genome. A second replication product migrates at approximately 9.5 Kb. These bands correspond to the two major AAV2 replication intermediates, referred to as replication form monomer (RF_M_) and dimer (RF_D_). The ratio of these intermediates appears to be the same for each of the constructs tested. Less DNA replication was detected in the pVM108 and pVM113 samples lanes than with pSub201(-). The replication defect is more pronounced with pVM108 and somewhat intermediate with pVM113. For each vector, similar amounts of DNA replication are detected at 24 hours and 48 hours.

An approximately 4 Kb band of replicated DNA is detected in the pVM108 sample lanes in Figure 3C that is not detected in the other lanes. The identity of this band is confirmed by the Southern analysis in Figure 3D. Digestion of the genomic DNA with ClaI indicates that the ~4KB replication product contains the backbone portion of pSub201(-). The marker lane in Figure 3D was made by combining separate PvuII and XbaI digestions of. pSub201(-). The migration of these bands can be used to determine that the ~4 Kb pVM108 replication product contains *AAVS1 *ITRs located between the PvuII and XbaI sites. The bands do not align exactly because the *AAVS1 *modified ITRs are slightly smaller than the wild type ITRs in pSub201(-).

### Site-specific integration

**Figure 4:**
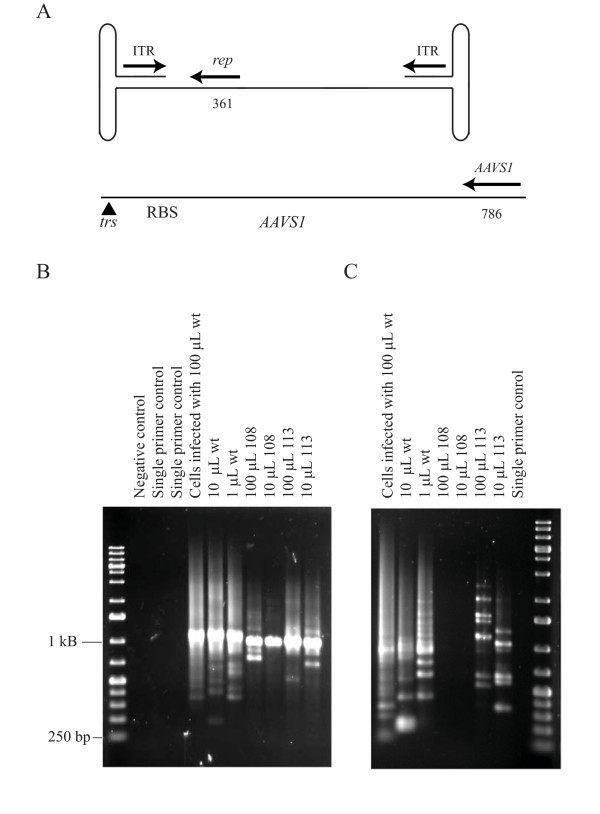
**Site-specific integration at *AAVS1***. (A) Diagrams (not to scale) of the AAV2 and *AAVS1 *primer pairs used in nested PCR assays. Each arrow represents a set of two nested primers used in a PCR assay. The number below the AAV2 *rep *primer set indicates the number of bases from the 5' end of the second primer to the end of the AAV2 genome. The number below the *AAVS1 *primer set indicates the number of bases from the *trs *to the 5' end of the second primer in the *AAVS1 *primer set. (B) Nested PCR assays using the *AAVS1 *primer set with the AAV2 *rep *gene primer set. The templates were genomic DNA isolated from HeLa cells infected with the indicated volumes of AAV2-containing supernatants produced using pSub201(-) (wt), pVM108 (108) or pVM113 (113). PCR products were resolved on a 1% agarose gel and stained with ethidium bromide. Single primer controls were done by using only the *AAVS1 *primer or the AAV2 primer in the second PCR. The template for the single primer controls was DNA from cells infected with 100 μl AAV2. (C) Nested PCR assays using the *AAVS1 *primer set with the AAV2 ITR primer set. The single primer control used only the AAV2 primer in the second PCR.

A nested PCR assay was used to detect integration at *AAVS1*. With this assay one primer set is designed to anneal to AAV2 and the second primer set is designed to anneal to *AAVS1*. The locations of the primer pairs that were used are diagramed on maps of AAV2 and *AAVS1 *in Figure 4A. Because the Rep primers and ITR primers are close to the ends of AAV2, the locations of the junctions within *AAVS1*can be estimated from the sizes of the PCR products (Fig. 4B and 4C). The major ~1 Kb bands in Figures 4B and 4C indicate a cluster of junctions in the area of the *AAVS1 trs *and RRS. This area of *AAVS1 *has been previously noted as a junction hotspot [40,44].

**Figure 5:**
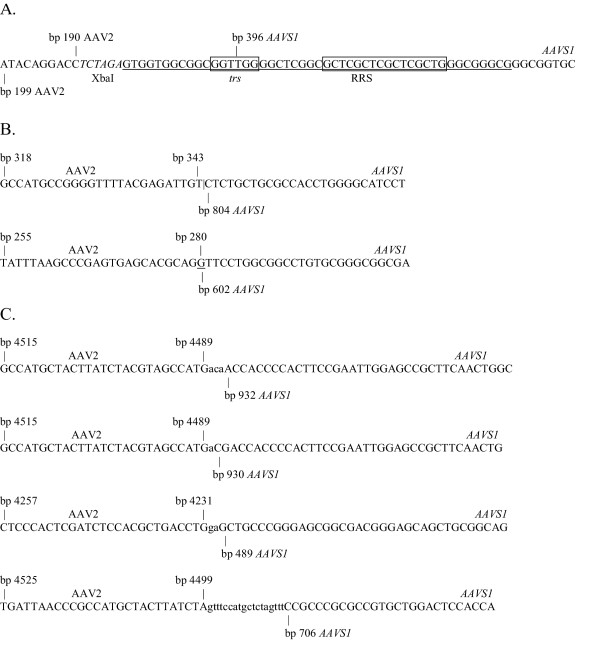
**DNA sequence analysis of the integration junctions**. Bases that are shared by *AAVS1 *and the packaged virus are underlined. (A) The sequence of the integration junctions detected in cells infected with virus made using the pVM108 or pVM113 constructs using a combination of the AAV2 *rep *primers and the *AAVS1 *primers in the integration assay. The XbaI site is indicated by italics. (B) The sequences of the integration junctions detected for the wild-type virus using a combination of the AAV2 ITR primers and the *AAVS1 *primers in the integration assay. (C) The sequences of the integration junctions detected for virus made using the pVM113 construct using a combination of the AAV2 ITR primers and the *AAVS1 *primers in the integration assay. Bases at the junctions that do not appear to belong to either sequence are indicated lower case. The last junction contains additional AAV2 homology that is not indicated in the figure.

Bohenzky et al. reported the conversion of a mutated ITR to the wild-type sequence, when only one ITR was modified [50,51]. Since the mechanism of this reversion is not entirely clear, we needed to eliminate this as a possibility for our vector in which both ITRs are mutated. Using a primer set specific for the wild-type ITR does not produce an amplification product for virus produced from pVM108 (Fig. 4C), demonstrating that the ITRs in pVM108 have not been converted to wild-type during virus production. Several integration junctions were cloned and sequenced. Figure 5A, B and 5C show the sequences that were recovered. The sequence in Figure 5A is the sequence of the integration junctions amplified from cells infected with virus produced from pVM108 and pVM113 using the AAV2 *rep *primers (Fig. 4A). All of the junctions amplified from cells infected by virus made from these constructs using the *rep *primers contain this sequence, which appears to be the modified ITR sequence joined to *AAVS1 *at the RRS/*trs *region. The sequences in Figure 5B were amplified from cells infected with wild-type virus using the ITR-specific primers. The sequences in Figure 5C were amplified from cells infected with virus produced from pVM113 using the ITR-specific primers. These results confirm that the PCR products shown in Figure 4 are AAV2-*AAVS1 *junctions.

## Discussion

AAV2 has a relatively low frequency of integration [9,52,53]. This is probably due to the fact that, unlike retroviruses, integration is not an obligatory part of the AAV2 life cycle. We and others have noted that the majority of AAV2/*AAVS1 *junctions occur at short regions of homology between AAV2 and *AAVS1 *[7,44]. We therefore hypothesized that increasing sequence homology between AAV2 and *AAVS1 *might increase either the frequency or site-specificity of AAV2 integration. One approach was to insert DNA sequences from *AAVS1 *into the AAV2 genome. The amount of sequence that can be added is limited by the packaging capacity of AAV2 [54]. Westarted with a modest insert of 49 bases in an area of AAV2 that would not interfere with replication or packaging. Our second approach was to expand an existing region of homology by replacing the RRS/*trs *region of the AAV2 ITRs with the corresponding region from *AAVS1*. This second strategy does not increase the size of the AAV2 genome, but there was concern that the *AAVS1 *sequence might not contain all of the sequence elements required for AAV2 replication and packaging.

We did not detect a marked increase in integration efficiency as indicated by the intensity of bands on our gels of PCR products (Fig. 4 and data not shown) with our modified viruses. We also failed to see a reproducible increase in integration site specificity, which would have been indicated by a reduced size range for the PCR products (Fig. 4 and data not shown). We interpret these results as indicating that the integration process is more similar to non-homologous end-joining than homologous recombination, even with the increased homology. This interpretation is consistent with the observations of Daya *et al*. who showed that DNA ligase IV and DNA PKcs can affect the ratio of *AAVS1 *to non-*AAVS1 *integration events by AAV2 [55]. It should be noted however that a small increase in the number of specific junctions mediated by the increased homology might have been masked by the natural clustering of junctions occurring in these areas.

Our results do indicate that the RRS and *trs *elements from *AAVS1 *and AAV2 are functionally interchangeable. A strand packaging bias was observed by Zhou et al [56] when they deleted 18 bases of one d-sequence in the context of a recombinant AAV2 vector plasmid containing a single modified ITR with two d sequences. Their interpretation of their data was that the deleted 18 bases contained a packaging signal. In our *AAVS1*-substituted ITR, these 18 bases are almost completely changed and/or deleted. We have previously demonstrated the existence of stable secondary structures in single-stranded versions of the sequences roughly centered on the AAV2 and *AAVS1 trs *[31]. We believe that these secondary structures, thought to be stem-loops, based on sequence analysis of multiple AAV serotypes [30], function as a critical packaging signal. An 18 base pair deletion of the d sequence would be predicted to destabilize the AAV2 stem-loop structure [30,31]. The 11 base sequence from *AAVS1 *which essentially replaces the 18 bases deleted by Zhou et al. [56] in our mutated ITR has only has 2, non-adjacent, bases of sequence identity with the wild-type AAV2 sequence (Fig. 1). It is therefore a reasonable inference that the stable secondary structure, the only other known commonality between the two sequences, is part of the packaging signal.

Having an intact *trs *as part of the packaging signal would have a selective advantage because it would prevent packaging of virus genomes in which the *trs *had been prematurely cleaved by Rep68/78 or cellular endonucleases. An intact *trs *is required for productive infection [14]. In the packaged, single-stranded form of the AAV2 genome, only the 3' end of the genome would have an intact *trs *stem-loop (Fig. 1). This *trs *stem-loop/packaging signal hypothesis is also consistent with the observations that the 3' end of the AAV2 genome enters the pre-formed capsid before the 5' end and that packaging appears to be driven by the 3' to 5' helicase activity of the Rep proteins [29,33,36,57,58].

A fundamental question in virology is centered on the origins of virus DNA sequences. The RRS/*trs *combination at the *MBS85 *gene (the *AAVS1 *locus in humans) has also been detected in mice and African green monkeys [59-62]. Although it cannot be formally ruled out that this sequence is the remnant of an AAV2 integration event that occurred prior to the rodent-primate evolutionary divergence, a more intriguing possibility is that the AAV2 origin of replication is derived from this genomic sequence.

One final concern is that the packaged virus that was believed to be modified may have been wild-type revertants. The integration assays shown in Fig. 4 make this possibility highly unlikely. Using AAV2 ITR primers designed specifically to detect the wild-type ITRs, we were not able to detect junctions when the virus with two modified ITRs was used to infect cells. In addition, we were able to clone and sequence junctions with *AAVS1 *that appear to have the modified ITR joined to *AAVS1 *(Fig. 5).

## Conclusions

The ability of these *AAVS1 *sequences to substitute for the AAV2 RRS and *trs *provides indirect evidence that the stable secondary structure encompassing the *trs *is part of the AAV2 packaging signal. These results also suggest a level of sequence flexibility that could promote rapid evolutionary divergence of AAVs.

## Methods

### Plasmids and modification of the AAV2 ITR

A synthetic ITR of the following sequence was supplied to us in a cloning vector by Blue Heron Biotechnology (Bothell, WA). 5'-TCT AGA GTG GTG GCG GCG GTT GGG GCT CGG CGC TCG CTC GCT CGC TGG GCG GGC GCG GGC GAC CAA AGG TCG CCC GAC GCC CGG GCT TTG CCC GGG CGC GCC CGC CCA GCG AGC GAG CGA GCG CCG AGC CCC AAC AGC TG-3'. This sequence and the ITRs in the AAV2 infectious clone pSub201(-) (a kind gift from Dr. R. Jude Samulski) are flanked by Xba I and Pvu II sites [63]. A subcloning strategy using these restriction sites was employed to replace either the left, right or both ITRs in pSub201(-) with the ITR synthesized by Blue Heron Biotechnology. As indicated in Table 1, pVM108 is a pSub201(-) derivative in which both ITRs are replaced with the ITR modified to match *AAVS1*. pVM113 is a pSub201(-) derivative in which the left ITR is replaced with the ITR modified to match *AAVS1*. pVM112 is similar to pVM113. pVM112 contains an additional 49 bp of *AAVS1 *DNA that was made by annealing the following oligonucleotides: 5'-CTA GAG CCT GGA CAC CCC GTT CTC CTG TGG ATT CGG GTC ACC TCT CAC TCC TTT ACT AGT-3' and 5'-CTA GAC TAG TAA GGA GTG AGA GGT GAC CCG AAT CCA CAG GAG AAC GGG GTG TCC AGG CT-3'. When annealed, these oligonucleotides produce 5' overhangs that are compatible with Xba I. The sequence was cloned into the Xba I site adjacent to the right ITR in pVM112. pVM141 is a pSub201(-) derivative in which the right ITR is replaced with the ITR modified to match *AAVS1*. pVM141 also contains the additional 49 bp of *AAVS1 *DNA present in pVM112. In pVM141 the DNA was cloned into the XbaI site adjacent to the left ITR. The sequence is in the same orientation in both vectors.

**Table 1:** Plasmid constructs used to make the modified viruses used in this study.

			^**a**^**Other**
	Left ITR	Right ITR	*AAVS1 *Insert
pSub201(-)	wt	wt	No
pVM108	AAVS1	AAVS1	No
pVM112	AAVS1	wt	Yes
pVM113	AAVS1	wt	No
pVM141	wt	AAVS1	Yes

^a^As described in the methods section, pVM112 and pVM141 contain an additional 49 bp of *AAVS1 *DNA.

### Transfection of HEK293 cells and preparation of virus supernatants

To produce virus, HEK293 cells (Stratagene) which contain the adenovirus E1 gene were co-transfected with the E1 deleted adenovirus helper plasmid pHelper (Stratagene) and the ITR-containing AAV2 plasmids using the calcium phosphate co-precipitation method. To perform this procedure, 250 μl of 2× HBS (280 mM NaCl, 1.5 mM Na_2_HPO_4_, 50 mM HEPES, pH 7.1) was added to a 250 μl volume of 0.5 M CaCl_2 _containing 14 μg pHelper and 14 μg of the AAV plasmid and immediately added to a 75 cm^2 ^flask of ~80% confluent Stratagene HEK293 cells that had been split 1:5 the previous day into DMEM media (Invitrogen) with 2 mM L-glutamine and 10% fetal bovine serum. After 2 days the cells were scraped from the plates, washed once with PBS and suspended in 0.5 ml cell lysis buffer (0.15 M NaCl, 50 mM Tris-HCl, pH 8.5). Cells were lysed by three cycles of freezing at -80°C and thawing. Unpackaged DNA was removed by adding 50 μl of Benzonase (Novagen) and incubating at 37°C for 2 hours.

### Determination of viral titers

For titration of packaged virus genomes, DNA was isolated from 25 μl of the virus supernatant. The volume was adjusted to 200 μl with a final concentration of 10 mM EDTA and 0.5% SDS. Next, 18.6 μg of proteinase K (Invitrogen) was added and the solution was incubated for 1 hr at 37°C. Proteins were removed by phenol-chloroform extraction. The DNA was ethanol precipitated with 10 μg of glycogen (Roche). The DNA was resuspended in 0.5 M NaOH, 1.5 M NaCl and hybridized to a positively charged nylon membrane (Hybond nucleic acid transfer membrane, GE Healthcare, Buckinghamshire, UK) using a dot blot apparatus. pSub201(-) contains an AAV2 genome flanked by Pvu II sites. A Pvu II digest of pSub201(-) was used as a standard. To probe the blot, pSub201(-) was digested with XbaI and ClaI. The 4310 bp XbaI fragment of the AAV2 genome from pSub201(-) was gel purified and random primer labeled using [α-^32^P]dCTP and oligolabeling beads (Ready to go DNA labeling beads, GE Healthcare, Buckinghamshire, UK). Hybrisol (Millipore, Temecula, CA) was used for the hybridization.

### Southern blotting analysis of virus replication

Total DNA from HEK293 cells transfected in parallel with those used for the preparation of virus supernatants was isolated using a DNeasy tissue kit (QIAGEN, Valencia, CA) 24 and 48 hours post transfection. Some samples were pre-treated with Dpn I to degrade input plasmid. Two micrograms of DNA from each sample was resolved on a 1% agarose gel and transferred to positively charged nylon membrane (Hybond nucleic acid transfer membrane, GE Healthcare, Buckinghamshire, UK) for Southern blotting analysis. Briefly, the DNA was first fragmented by soaking the gel in several volumes of 0.25 M HCl for 10 minutes. The gel was washed for several minutes with water and DNA was denatured by soaking the gel in 1.5 M NaCl, 0.5 N NaOH for 30 minutes. The gel was placed front down on a solid support covered by a piece of Whatman 3 mm paper long enough to drape into a reservoir of 10× SSC (KD Medical, Columbia, Maryland). The positively charged nylon membrane was placed on the back of the gel below a stack of paper towels. After a 16 hour transfer the membrane was washed several times with 5× SSC and dried in bright light. The membrane was probed with random primer labeled pSub201(-). The pSub201(-) probe was made using [α-^32^P]dCTP and oligolabeling beads (Ready to go DNA labeling beads, GE Healthcare, Buckinghamshire, UK). Hybrisol (Millipore, Temecula, CA) was used for the hybridization. A 1 Kb DNA ladder (Fermentas, Glen Burnie, MD) and pSub201(-) were used as DNA markers. Several of the bands in the 1 Kb DNA ladder contain DNA that is in pSub201(-) and hybridize to the probe. The pSub201(-) marker was made by combining PvuII and XbaI digests of pSub201(-). The digestions were stopped with 5 mM EDTA before combining.

### Integration assays

A 25 cm^2 ^flask of ~25% confluent HeLa cells was infected for 2 hours in medium without fetal bovine serum (FBS). After 2 hours the medium was replaced with medium containing 10% FBS. Cells were harvested 48 h after infection, and genomic DNA was isolated using a DNeasy tissue kit (QIAGEN, Valencia, CA). Several combinations of AAV2 and *AAVS1 *primer pairs were used to detect integration by nested PCR. For the nested PCR assay 50 ng of genomic DNA and 100 ng of each primer in a 50-μl reaction volume were used in the first round of PCR amplification. After an initial incubation for 4 min at 94°C, the reaction mixture was subjected to 28 cycles of PCR amplification for 1 min at 94°C, 1 min of annealing at 63°C, and 3 min at 72°C, using FastStart DNA polymerase (Roche). One percent of the amplification product was diluted into a new reaction mixture containing the second pair of primers. The PCR parameters were the same as those for the first amplification. The following primer sets were used. In each set the first primer listed was used in the first amplification and the second primer was used in the second amplification. AAV2 rep 5'-CAC CCA GTT CAC AAA GCT GTC AGA AAT G-3' and 5'-TCG CTG GGG ACC TTA ATC ACA ATC TC-3', AAV2 cap 5'-CAG GAC AGA GAT GTG TAC CTT CAG GG-3' and 5'-TGG ACA CTA ATG GCG TGT ATT CAG AGC-3', AAV2 ITR 5'-GCC TCA GTG AGC GAG CGA G-3' and 5'-GCA GAG AGG GAG TGG CCA-3', *AAVS1 *5'-AGG CAG ATA GAC CAG ACT GAG CTA TGG-3' and 5'-CAG GGA AGG AGA CAA AGT CCA GGA-3'. PCR products were resolved on a 1% agarose gel and stained with ethidium bromide. Cloning and sequencing of PCR-amplified junctions were performed as described previously [44].

## Declaration of Competing interests

R.A.O. is a co-inventor on several patents involving AAV vectors. To the extent that this work will increase the value of those patents, he has a competing interest.

## Authors' contributions

VJM was the primary contributor to project conception, overall experimental design, plasmid construction, virus production, cell infection, integration assays, data analysis and writing of manuscript. He also performed all experiments.

RAO was overall project coordinator, and contributed to experimental design, data analysis and writing of the manuscript.

All authors read and approved the final manuscript.
